# Slow Subcutaneous Release of Glatiramer Acetate or CD40-Targeting Peptide KGYY_6_ Is More Advantageous in Treating Ongoing Experimental Autoimmune Encephalomyelitis

**DOI:** 10.3390/neurolint16060114

**Published:** 2024-11-20

**Authors:** Gisela M. Vaitaitis, David H. Wagner

**Affiliations:** Webb-Waring Center, Department of Medicine, University of Colorado, Anschutz Medical Campus, 12850 E Montview Boulevard, Aurora, CO 80045, USA; gisela.vaitaitis@cuanschutz.edu

**Keywords:** multiple sclerosis, glatiramer acetate, CD40, drug delivery, slow-release

## Abstract

Background/Objectives: One of the first-line disease-modifying treatments of multiple sclerosis (MS) is Glatiramer Acetate (GA), which requires daily or three-times-weekly subcutaneous injections. Disease progression, while slowed, still occurs with time. Increasing the impact of the treatment while decreasing the frequency of injections would be ideal. The mechanism of action of GA remains undefined. We developed an alternate approach, KGYY_6_, whose mechanism of action targets the CD40 receptor with promising results in an Experimental Autoimmune Encephalomyelitis (EAE) model. Methods: GA and a CD40-targeting peptide, KGYY_6_, were formulated as slow-release particles used to treat EAE in C57BL/6 mice. Results: Compared to liquid formulations, the particle formulations vastly improved drug efficacy in both cases, which would be advantageous in treating MS. GA is a combination of randomly generated peptides, in the size range of 5000–9000 Da, using the amino acids E, A, Y, and K. This approach introduces batch differences that impacts efficacy, a persistent problem with GA. KGYY_6_ is generated in a controlled process and has a motif, K-YY, which could be generated when manufacturing GA. When testing two different lots of GA or KGYY_6_, the latter performed equally well across lots, while GA did not. Conclusions: Slow-release formulations of both GA and KGYY_6_ vastly improve the efficacy of both, and KGYY_6_ is more consistent in efficacy across different lots.

## 1. Introduction

Multiple sclerosis (MS) is a neurodegenerative autoimmune disease of the central nervous system (CNS) with varying symptoms including fatigue, vision disturbance, weakness, cognitive disability, and eventual paralysis. The autoimmune etiology of the disease was elucidated from studies using the Experimental Autoimmune Encephalomyelitis (EAE) mouse model and from cellular analysis of human brain and spinal cord lesions. Inflammatory cells from the periphery infiltrate the CNS [1,2,3,4,5] contributing to disease development. A subset of helper T cells, CD40-expressing CD4 T cells (Th40), emerged as primary drivers of the disease [6]. Isolated Th40 cells cause a more severe form of EAE than conventional CD4^+^ T cells in disease transfer models, confirming pathogenic effector status [6]. Additionally, in disease transfer studies, conventional CD4^+^ T cells expressed CD40 after disease development [6].

CD40 interaction with CD154 is an inflammation checkpoint prominent in many autoimmune diseases including MS [7,8]. Th40 cells are significantly (*p* < 0.001) elevated in peripheral blood from Relapsing Remitting MS (RRMS) patients [9], as well as secondary and primary progressive MS patients [9]. Th40 cells were detected in the CSF of MS patients, demonstrating the ability to breach the CNS [9]. The therapeutic importance of CD40-CD154 signaling block using monoclonal antibodies was demonstrated in EAE [10]. Therapeutic attempts to block CD40-CD154 interaction using monoclonal antibodies Toralizumab [11] and, later, Frexalimab [12] in MS were developed. The Toralizumab study dosed patients between 1999 and 2001 and patients were followed for 5 years after the last dose; the study was completed in 2006 [11]. However, while the drug demonstrated good safety and some immunological shift toward an anti-inflammatory profile, the results were not reported until 2021 [11], and further clinical advances with Toralizumab since 2006 have not been reported. Frexalimab is advancing to phase 3 trials with promising outcomes in the phase 2 clinical trial. An early issue with the anti-CD154 approach was the development of thrombotic emboli [13,14], but the recent version, Frexalimab, included alteration of the Fc portion that did not result in emboli [15], suggesting that antibody Fc portion plays a role in thrombosis.

To circumnavigate antibody issues, small peptides were created that target CD40 [16,17] in combination with specific integrins [18]. The peptides target CD40 on multiple cell types, including Th40, pathogenic effector cells in EAE [17], and MS [9] to modulate rather than block CD40-CD154 signaling [18]. This is an important distinction from antibodies that block signaling and can deplete the target cells to cause immune suppression. With the CD40-targeting peptides, rather than cell depletion, the CD40-CD154 signaling is simply modulated to favor a less inflammatory outcome [16,17,18]. A major advantage is that, unlike antibodies, small peptides penetrate the blood–brain barrier. The CD40-targeting peptide KGYY_6_ delayed disease onset and prevented severe symptoms in EAE [17]. The mechanism of action of KGYY_6_ in EAE was to prevent the trafficking of Th40 and other T cells by increasing CD69-mediated retention in the lymph nodes, as well as promoting an increase in the production of the anti-inflammatory cytokine IL-10 [17]. Another version of the peptide, KGYY_15_, prevented Type 1 Diabetes in a mouse model by preventing the expansion of Th40 cells [16]. However, in the EAE model, it is not possible to assess the prevention of Th40 cell expansion since the actual disease induction regimen (the complete Freund’s adjuvant component) expands Th40 cells non-specifically [6]. KGYY_6_ is composed of alanine (A), lysine (K), Glycine (G), and tyrosine (Y) with the sequence AKKGYY. That sequence contains the known CD40-interacting motif K-YY and has been demonstrated to bind both Th40 and memory T cells in EAE [17], but likely would bind other CD40-bearing cells as well.

One of the first-line disease-modifying treatments in MS is the drug Copaxone or Glatiramer Acetate (GA) [19]. Treatment of RRMS subjects with GA resulted in significant reductions in CNS lesions [20] and reduced annual relapse rate by up to 30% [21]. Because of its safety profile, relative efficacy early on, cost-effectiveness, and ease of use (self-injected subcutaneously), GA became a prominent first-line treatment for MS [22,23,24,25]. GA is a mixture of randomly arranged polypeptides consisting of glutamic acid (E), alanine (A), tyrosine (Y), and lysine (K). The average polypeptide molecular weight is 5000–9000 Da and the molar fractions of E, A, Y, and K residues are 0.129–0.153, 0.392–0.462, 0.086–0.100 and 0.300–0.374, respectively (NIH; National Library of Medicine). The mechanism of action is unclear but is thought to involve GA-specific regulatory T cells and a Th1-Th2 shift to suppress auto-aggressive Th1 cells [25,26,27]. GA may also influence monocytes and dendritic cells [25,26,27]. With the exception of E, GA and KGYY_6_ contain the same amino acids; the K-YY motif is established in KGYY_6_ and can occur randomly in GA.

GA is injected daily with a formulation of 20 mg/mL or 3 times weekly at 40 mg/mL. This is a long-term treatment and while the annual relapse rate is reduced, over time, disease progression resumes. Adverse events include injection site rash, lipoatrophy, dyspnea, and chest pain, thus minimizing the number of injections would be ideal. To that end, KGYY_6_ and GA were formulated into particles designed for slow release utilizing Poly(lactic-co-glycolic acid) (PLGA), an FDA-approved biodegradable copolymer [28]. PLGA particles degrade by hydrolysis, thereby eliciting the sustained release of the drug. The resulting lactic and glycolic acids are then eliminated through normal metabolic pathways [28].

Here, we demonstrate that KGYY_6_ or GA formulated as PLGA particles and delivered subcutaneously (s.c.) perform vastly better than liquid formulations, delivered s.c., to prevent severe symptoms of EAE. This is accomplished while injecting less frequently than is recommended for currently available GA and suggests a novel approach for first-line MS drug delivery. We also reveal a dramatic batch variation with GA that was not seen with KGYY_6_.

## 2. Materials and Methods

### 2.1. Mice

C57BL/6 mice (Taconic Biosciences, Germantown, NY, USA; MPF colony) were housed at the University of Colorado Anschutz Medical Campus, an AAALAC-approved facility. Experiments were performed under an IACUC-approved protocol (#00214) that adhered to the NIH Public Health Service Policy on Humane Care and Use of Laboratory Animals.

### 2.2. KGYY_6_ Peptide and GA

Two lots of KGYY_6_ peptide, acetyl-AKKGYY-amide (GenScript, Piscataway, NJ, USA), was supplied as an acetate salt that was dissolved in PBS and used in liquid formulation treatments or was made into PLGA particles (see Section 2.3). GA (Teva Pharmaceuticals USA, Parsippany, NJ, USA; Copaxone; NDC68546-325-12; S/N 10085101263195; Lot #111438 (Lot #1 in manuscript) and Lot #14424 (Lot #2 in manuscript)) was used as liquid formulation or was made into PLGA particles (see Section 2.3).

### 2.3. PLGA Particle Formulation

PLGA (Acros Organics; code 436200050) and ethyl acetate (Fisher Chemicals; E145-500) were from Thermo Fisher Scientific, Waltham, MA, USA. Vitamin E TPGS (PN: TG0101NF) was from Antares Health Products, Inc., St. Charles, IL, USA. Trehalose was from Sigma, St. Louis, MO, USA. KGYY_6_ peptide and GA were as detailed in Section 2.2. PLGA particles were prepared as described [29] using the KGYY_6_ peptide in PBS at 50 mg/mL or GA as supplied by the manufacturer at 40 mg/mL with the addition of 20× PBS to achieve a final concentration of 1× PBS. Final particles were resuspended in PBS containing 12.5 mg/mL Trehalose as a cryoprotectant and were frozen at −20 °C until use. Unused, thawed particles were stored at 4 °C for up to 1 week. A typical batch of GA PLGA particles had a 42–48% encapsulation rate and a concentration of 4.2–4.4 mg GA per ml of resuspended particles. A typical batch of KGYY_6_ PLGA particles had a 20–24% encapsulation rate and a concentration of 4.2–4.8 mg KGYY_6_ per mL of resuspended particles. The size and quality of the particles were determined using a ZetaSizer instrument from Malvern Panalytical, Westborough, MA, USA.

### 2.4. Peptide Release from PLGA Particle

PLGA particles in PBS/Trehalose were aliquoted and stored at 37 °C for up to 20 days. At various times, aliquots were removed, and particles pelleted. The protein content was measured in the supernatant using a Bio-Rad Protein Assay (Cat# 5000006; Bio-Rad, Hercules, CA, USA). This assessment was only possible for GA as KGYY_6_ is too small to register.

### 2.5. EAE Induction, Treatment, and Disease Scoring

Female and male 10–12 week-old C57BL/6 mice were immunized subcutaneously on the upper back/neck with 100 µL of an emulsion of MOG35-55 peptide (50 µg in 50 μL PBS) and complete Freund’s adjuvant (CFA; 75 µg M. Tuberculosis H37 RA in 50 μL incomplete Freund’s adjuvant (mineral oil)), followed by intraperitoneal pertussis toxin (200 ng in 100 µL PBS) injections on days 0 and 2 (for details, see Figure S1). Mice were randomly assigned to treatment cohorts (*n* = 10 in all cohorts) or a vehicle control cohort (*n* = 30 in particle studies and *n* = 40 in liquid studies). Some cohorts received s.c. injections of liquid KGYY_6_ peptide either at 4 or 8 mg/kg, in 100 µL PBS, 3 times weekly. Other cohorts received s.c. injections of liquid GA at either 0.6 (3 times weekly) or 100 mg/kg (5 times weekly) as supplied by the manufacturer or diluted in PBS for the lower dose. Some cohorts received KGYY_6_ PLGA particles s.c. at a dose of 8 mg/kg with respect to peptide, every 3–4 days. Yet, other cohorts received GA PLGA particles s.c. at either 4 or 8 mg/kg with respect to GA, every 3–4 days. All treatments started on day 6 after EAE induction. Vehicle control mice received injections of PBS. All mice were monitored daily for disease and scored on a scale from 0 to 5: 0—no abnormalities; 0.5—clutching hind limbs or hesitation; 1—limp tail or weak hind limbs or wobbly gait; 1.5—limp tail and clutching hind limbs; 2—limp tail and weak hind limbs and/or wobbly gait. A mouse supports and propels itself using its hind limbs. A mouse does not immediately right itself when placed on its back; 2.5—limp tail and very weak hind limbs and/or wobbly gait. A mouse is barely able to support and propel itself using hind limbs; 3—limp tail and one weak hind limb, while the other is completely paralyzed. Mouse still uses the weak hind limb to somewhat propel itself; 3.5—limp tail and one weak hind limb, while the other is completely paralyzed. A mouse does not use the weak limb, which paddles but is almost at paralysis; 4—limp tail and complete hind limb paralysis. Often a mouse displays spastic hypertonia and involuntarily crosses its hind limbs; 4.5—complete paralysis of the hind quarter and weak fore limb(s); 5—moribund. Mice that reached a level 4.5 or 5 and/or lost more than 15% of their body weight that was not regained within 2–3 days were immediately euthanized. The data are reported as the mean daily clinical score for all animals in each group.

### 2.6. Statistics and Graphs

Statistics and graphs were generated using GraphPad Prism 10.4.0 (GraphPad Software, Boston, MA, USA).

## 3. Results

### 3.1. KGYY_6_ Delivered s.c. as a Liquid Formulation TRENDS Toward an Impact on EAE Disease While GA Does Not

**Figure 1:**
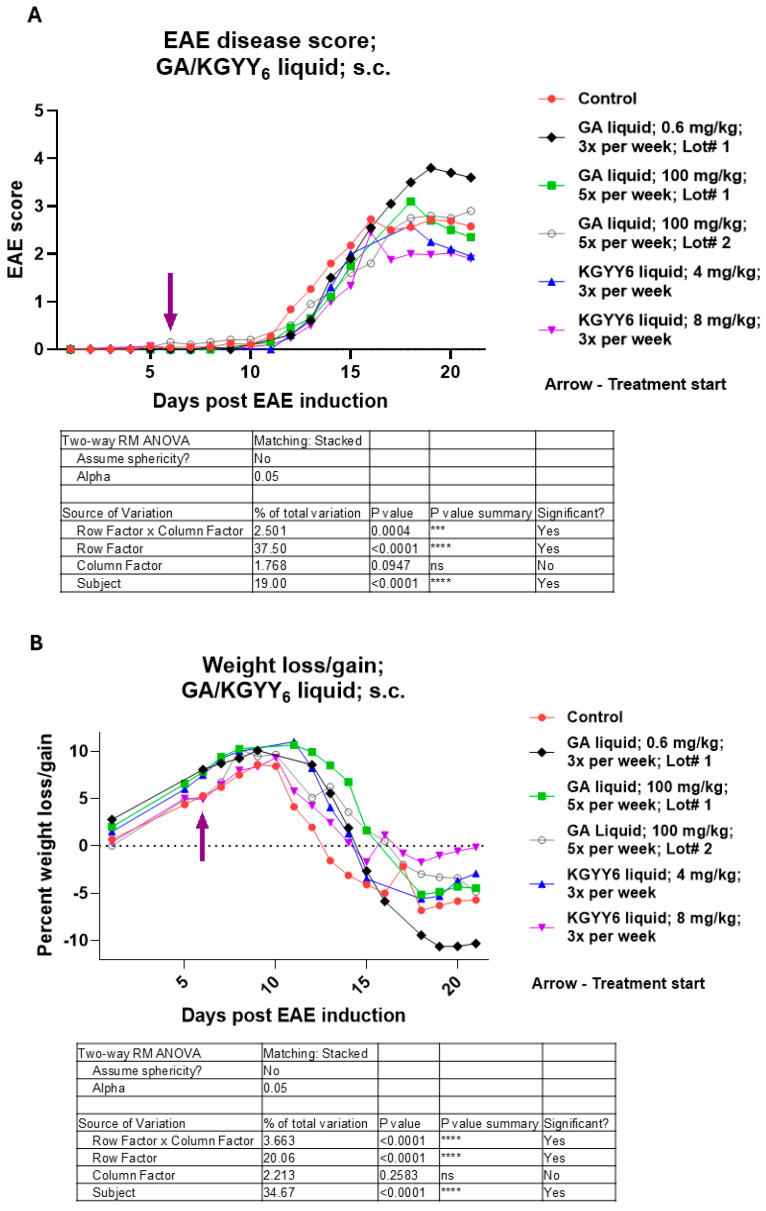
Liquid formulations of KGYY_6_ or GA do not significantly ameliorate EAE. Mice were EAE-induced and treated with liquid KGYY_6_ or GA at indicated dosages, starting on day 6 post-induction. (**A**) Severity of disease was scored and (**B**) weight loss/gain determined. Tables show *p*-values from Two-way ANOVA analysis for each graph. The data in the graphs are displayed using all available data points. However, since not all groups had data points for the exact same days, only those days that had data points in common for all cohorts in the graph were used when performing Two-way ANOVA with Tukey’s multiple comparisons. ***—*p* < 0.001; ****—*p* < 0.0001; ns—not significant.

KGYY_6_ peptide has been demonstrated to prevent severe symptoms in EAE when delivered intravenously (i.v.) with three infusions during one week, starting on the first day of symptoms, and with no additional boosters [17]. From a human therapeutic perspective, i.v. administration is not ideal, requiring regular visits to an infusion center. The current route of administration of GA is s.c.; therefore, the efficacy of KGYY_6_ compared to that of GA by s.c. route was examined. We tested two different lots of GA. Treatment was administered starting on day 6 post-disease induction when there were no visible symptoms, but the disease course is well underway. Two doses of GA Lot #1 were tested: 0.6 mg/kg, the approximate human dose, and 100 mg/kg, a dose that was required to ameliorate EAE disease in previous mouse studies [30,31]. In the current study, GA Lot #1 was not efficacious in ameliorating EAE symptoms at either dose level compared to control; however, both doses slightly delayed disease onset (Figure 1A). We tested a second lot of GA at the higher dose level, but it also showed no efficacy (Figure 1A; Lot #2). Two doses of KGYY_6_ in PBS, 4 and 8 mg/kg, were tested. As with GA, KGYY_6_ delayed disease onset slightly (Figure 1A). Starting on day 17, the 8 mg/kg dose cohort had an EAE score that was 0.6 points lower than the control and, on the last day, almost 0.7 points lower; however, the difference did not reach significance (Figure 1A). On day 21 for the 4 mg/kg KGYY_6_ dose, while not significant, there was likewise a 0.7-point difference in the EAE score compared to the control (Figure 1A).

In the EAE model, weight loss correlates directly with disease severity. When weight loss/gain was assessed, there was a delay in weight loss in the GA Lot #1 at 100 mg/kg cohort compared to control on days 11 through 15 (Figure 1B). The onset of weight loss was also somewhat delayed in all the other treated cohorts compared to the control (Figure 1B). Interestingly, the 8 mg/kg KGYY_6_ cohort trended toward less weight loss than the other cohorts.

### 3.2. Particle Formulation of KGYY_6_ and GA

**Figure 2:**
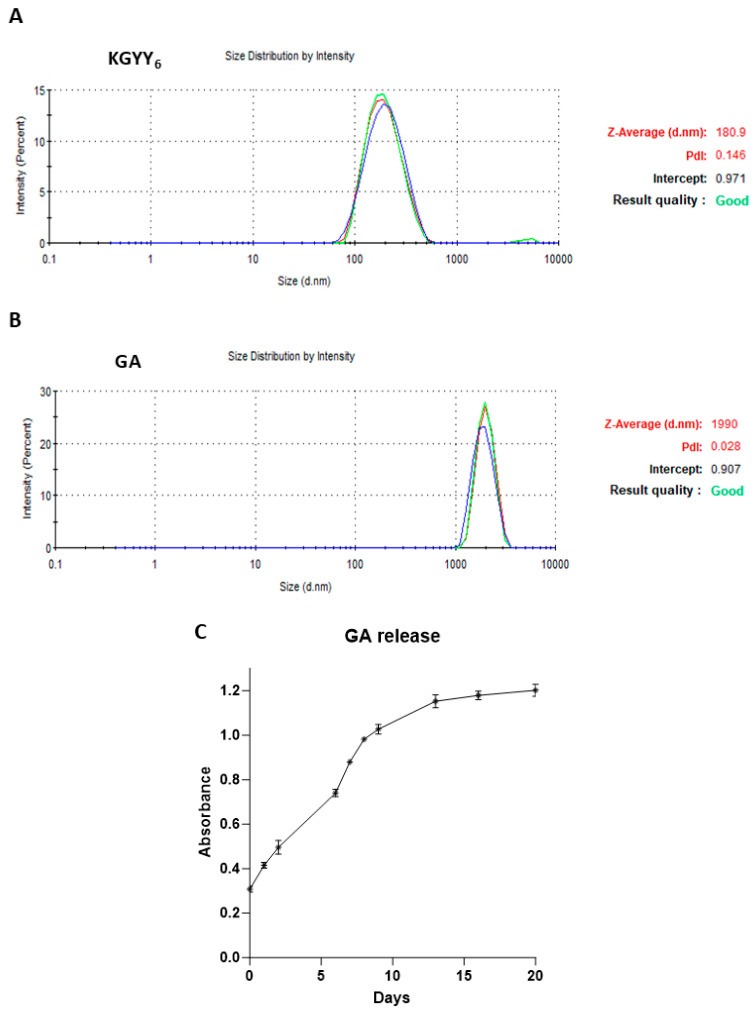
KGYY_6_ and GA PLGA particles. PLGA particles were generated and the size and quality were determined for (**A**) KGYY_6_ particles and (**B**) GA particles. Red, green and blue lines represent three different assessments of the particles. (**C**) The resulting GA particles were subjected to in vitro peptide release studies at 37 °C over time. Slow-release absorbance for GA is depicted.

Encouraged by the trend to lower disease scores and minimize weight loss in the s.c. liquid trials, we speculated that a slow-release formula may work better than a liquid bolus. We attempted to encapsulate KGYY_6_ in chitosan nanoparticles. However, with several different chitosan formulations, the encapsulations failed. Therefore, we employed PLGA. Utilizing a double emulsion PLGA particle preparation technique [29], KGYY_6_ and GA were encapsulated. Encapsulation of the 770 Da KGYY_6_ peptide formed nanoparticles with an average diameter of 180 nm and a polydispersity index of 0.146 (Figure 2A). The KGYY_6_ encapsulation rate was 20–24%. Encapsulation of GA, which has a molecular weight of 5000–9000 Da, resulted in microparticles with an average diameter of 1990 nm, a polydispersity index of 0.028 (Figure 2B), and an encapsulation rate of 42–48%. Both KGYY_6_ and GA particles were easily drawn into syringes fitted with 25-gauge needles and stayed well suspended during handling and injections into animals. Typical in vitro release of GA when the PLGA particles are stored at 37 °C is shown, with steady release over two weeks, which then plateaued (Figure 2C). Release assessment for KGYY_6_ particles was not possible because the small size of the peptide is outside the limitations of the protein assay.

### 3.3. Slow Release of KGYY_6_ or GA Ameliorates EAE Significantly

**Figure 3:**
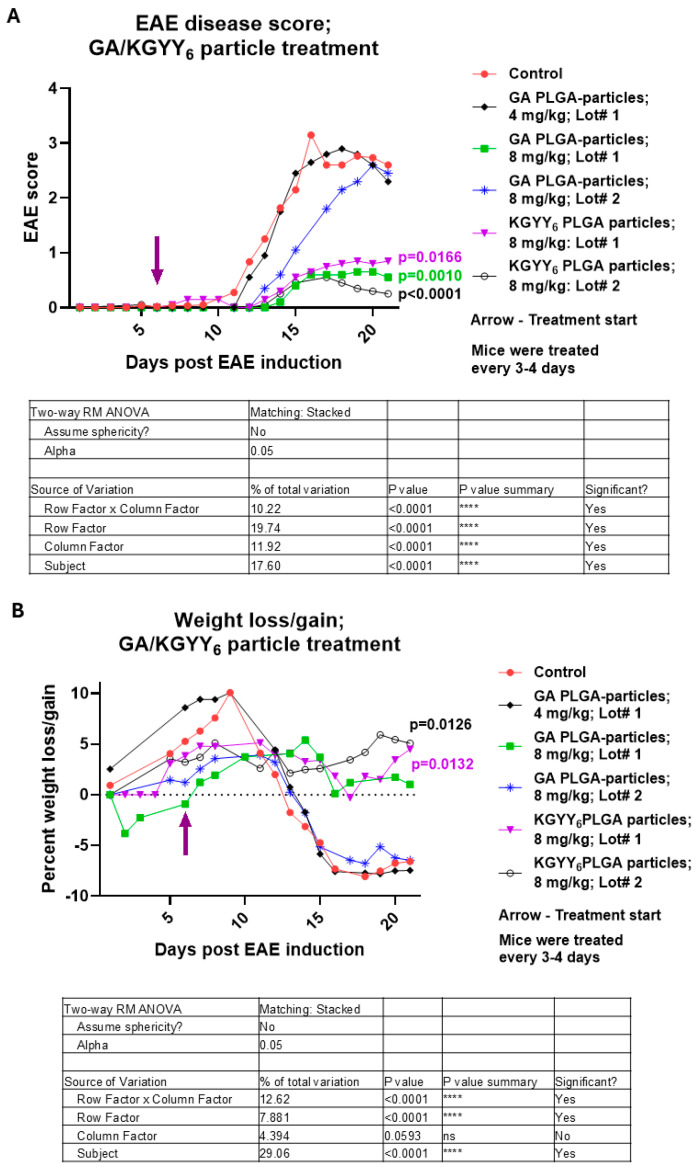
PLGA particle formulations of KGYY_6_ and GA significantly ameliorate EAE. Mice were EAE-induced and treated with KGYY_6_ or GA PLGA particles at indicated dosages, starting on day 6 post-induction. (**A**) Severity of disease was scored and (**B**) weight loss/gain determined. Tables show *p*-values from Two-way ANOVA analysis for each graph. *p*-values in graphs are from Tukey’s multiple comparisons test. The data in the graphs are displayed using all available data points. However, since not all groups had data points for the exact same days, only those days that had data points in common for all cohorts in the graph were used when performing Two-way ANOVA with Tukey’s multiple comparisons. ****—*p* < 0.0001; ns—not significant.

To assess how slow release of the treatment peptides could impact EAE, KGYY_6_ or GA PLGA particles were injected s.c. into EAE-induced mice on day 6 post-induction. Treatment with GA Lot #1 in the slow-release formulation was carried out at two different doses, 4 or 8 mg/kg. While the lower dose group had EAE scores that were not different from control mice, the higher dose of GA significantly ameliorated disease scores (Figure 3A; GA PLGA particles Lot #1; *p* = 0.0010). Not only were symptoms significantly delayed, but symptom severity was significantly reduced by more than 2 points compared to the control (Figure 3A). Using the GA Lot #1 treatment formulation at 8 mg/kg, symptoms only reached a high score of 0.65 and ended at 0.55 (Figure 3A). We tested GA Lot #2 as a PLGA particle formulation as well, and while it delayed disease onset, it eventually failed to ameliorate disease (Figure 3A; GA Lot #2). Two different lots of KGYY_6_ formulated as PLGA particles were tested. KGYY_6_ Lot #1 at 8 mg/kg performed as well as GA PLGA particles Lot #1 at ameliorating disease (Figure 3A; KGYY_6_ PLGA particles Lot #1, *p* = 0.0166). Treated mice only achieved a score of 0.85 at the highest. Additionally, like the GA PLGA particle Lot #1 treatment, the onset of low-grade symptoms was delayed by KGYY_6_ Lot #1 PLGA particle treatment. We tested a second lot of KGYY_6_, Lot #2, formulated as PLGA particles at a dose of 8 mg/kg and found that this lot performed equally well, reaching a high score of 0.55 and ending at 0.25 (Figure 3A; KGYY_6_ PLGA particles Lot #2; *p* < 0.0001).

When weight loss/gain was assessed, KGYY_6_ particle treatment, utilizing both lots at 8 mg/kg, clearly prevented the weight loss observed in the untreated control mice (Figure 3B; KGYY_6_ PLGA particles; Lot #1—*p* = 0.0132, Lot #2—*p* = 0.0126). GA Lot #1 at 8 mg/kg did not significantly impact weight loss relative to the control but trended toward prevention of weight loss (Figure 3B; GA PLGA particles Lot #1). GA Lot #1 PLGA particles at 4 mg/kg did not prevent weight loss and neither did GA Lot #2 (Figure 3B; GA PLGA particles Lot #1, 4 mg/kg, and GA PLGA particles Lot #2).

### 3.4. Slow-Release Formulations Perform Better than Liquid Formulations

**Figure 4:**
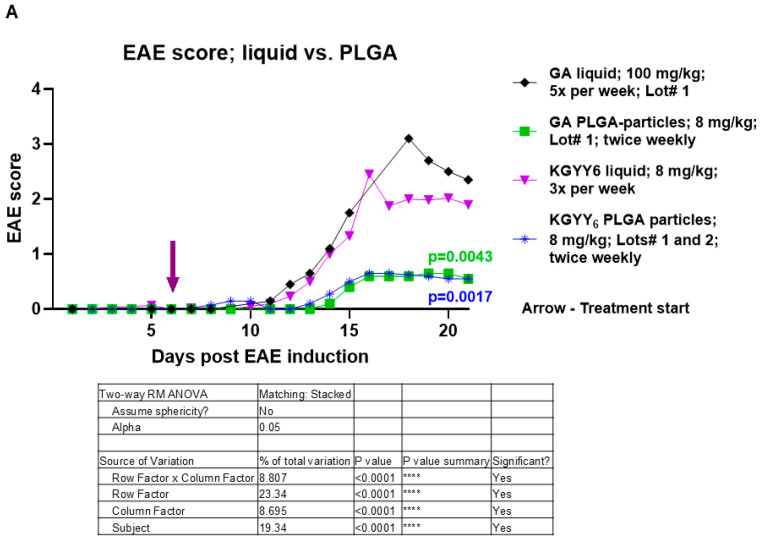

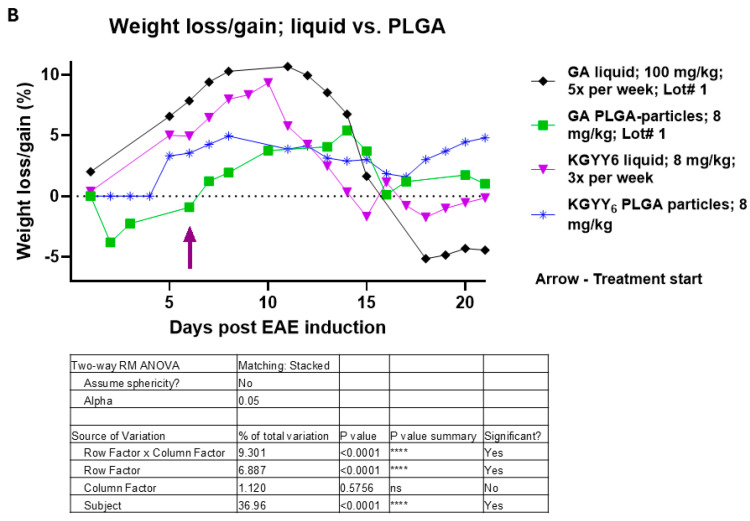
PLGA particle formulations are significantly better than liquid formulations at ameliorating EAE. Results from liquid- and PLGA-treated mice were directly compared for (**A**) EAE score and (**B**) weight loss/gain. Tables show *p*-values from Two-way ANOVA analysis for each graph. *p*-values in A are from Tukey’s multiple comparisons test. The data in the graphs are displayed using all available data points. However, since not all groups had data points for the exact same days, only those days that had data points in common for all cohorts in the graph were used when performing Two-way ANOVA with Tukey’s multiple comparisons. ****—*p* < 0.0001; ns—not significant.

While the data clearly demonstrates that slow-release formulations performed better than liquid formulations in ameliorating EAE, we performed direct comparisons of the data from the slow-release studies and the liquid studies. Only Lot #1 was considered for GA, while KGYY_6_ Lots #1 and 2 were considered together for KGYY_6_ PLGA particles, as the liquid KGYY_6_ lot was an entirely different lot. GA Lot #1 PLGA particles were significantly better than the same in liquid form at ameliorating EAE scores (Figure 4A; compare GA liquid Lot #1 and GA PLGA particles Lot #1; *p* = 0.0043). KGYY_6_ combined PLGA particle lots were significantly better than the liquid KGYY_6_ at ameliorating EAE scores (Figure 4A compare KGYY_6_ PLGA particles and KGYY_6_ liquid; *p* = 0.0017). When assessing weight loss/gain, while PLGA particles trended toward less weight loss, there was no significant difference between PLGA particles and liquid formulations for either GA or KGYY_6_ (Figure 4B).

### 3.5. Adverse Events

Over 2 weeks, in the GA PLGA particle Lot #1 cohort at 8 mg/kg, four of the five male mice developed skin lesions around the injection sites from the particle injections. None of the five females in that group developed lesions. No adverse events were observed in the GA PLGA particles Lot #1 at 4 mg/kg or in GA PLGA particles Lot #2. Likewise, no adverse events were observed in any of the KGYY_6_ PLGA particle cohorts.

## 4. Discussion

An issue with therapeutic approaches includes delivery formulation. Current treatment parameters for GA involve daily injections or three-times-weekly injections at increased dosage. Here, we provide proof of concept that a slow-release formulation has superior clinical outcomes using the EAE model. This finding was true for GA and for KGYY_6_, which clearly were effective at ameliorating EAE symptoms when formulated as PLGA particles and administered s.c. every 3–4 days. We surmise that this improved effect, compared to liquid formulations s.c., is due to the steady, slow release of treatment drug, rather than a bolus that is quickly cleared. Our in vitro assays studying GA release from the particles support this since peptide content in the supernatant increased over time at 37 °C and the release was steady for at least two weeks. This indicates that administration less frequently than every 3–4 days is likely possible while maintaining efficacy. However, this would require verification by pharmacokinetic studies in vivo.

Weight loss generally mirrors the disease score in EAE, but the EAE score appears to be a more precise measurement of disease severity than weight loss. There was no significant difference in weight loss between liquid- and PLGA particle-treated mice for both GA and KGYY_6_. This is likely due to that, while liquid formulations did not significantly impact weight loss compared to the control, the weight loss was still trending to be less than the control. Therefore, those numbers were closer to the decreased weight loss seen with the particle formulations and when performing the direct comparison between liquid and particle formulations; consequently, there was no statistical difference.

GA is manufactured by a process that generates random peptides composed of E, K, A, and Y between 5000 and 9000 Da. The KGYY_6_ peptide contains an amino acid motif (K-YY) that was shown to interact with CD40 [16]. Interestingly, this motif could be produced randomly by the amino acids in the process that generates GA. Therefore, one of the modes of action of GA may include modulation of CD40 signals in the same way that KGYY_6_ modulates those signals. That modulation could occur on any given cell bearing CD40, including Th40 cells, macrophages, and B cells.

Because of the randomness of producing GA, it is inherently difficult to control lot-to-lot differences. We suggest that KGYY_6_, with the known sequence AKKGYY, is a lot-controlled treatment drug due to its determinate sequence and non-random synthesis. The current data bear this scenario out as Lot #1 of GA, formulated as PLGA particles, was excellent at ameliorating EAE, but a second GA lot was not, while two different lots of KGYY_6_, formulated as PLGA particles, worked equally well at ameliorating disease. In addition, KGYY_6_ worked as well as GA Lot #1.

As PLGA particles break down, free lactic and glycolic acids are released and enter normal metabolic pathways. However, lesions on 4 of 30 mice that received the GA PLGA particles were noted and, interestingly, occurred only in male mice. The 20 KGYY_6_ PLGA particle-treated mice displayed no lesions. We speculate that lesion development is due to the larger size of GA PLGA particles compared to the KGYY_6_ PLGA particles. Because of the size difference, the particles would necessarily traffic and interact differently in the subcutaneous space. It is possible that the smaller particles are able to traffic through the capillary bed and/or are able to clear the space by interacting/being taken up by cell types such as macrophages and dendritic cells [32]. The larger particles may not be able to traffic and thus remain in the subcutaneous space to hydrolyze at the injection site. Hydrolysis of PLGA releases lactic and glycolic acids, creating a strongly acidic microenvironment, which, in turn, would cause lesions [33]. There is no clear reason for the sex difference in the occurrence of lesions, but it is known that there are differences in subcutaneous lipid content between males and females and it is speculated that this can impact drug delivery [34]. We speculate that such differences may impact how carriers such as PLGA interact with the subcutaneous space as well and that males are more sensitive to certain sizes of PLGA particles. We were not successful in encapsulating KGYY_6_ in several formulations of chitosan, but there are several other types of nanoparticles, and it is highly likely that if one of those successfully can encapsulate peptide, lesions may be avoided.

The present data serve as proof of concept that a slow-release mechanism improves disease scores while decreasing the injection frequency. Since no adverse events were associated with KGYY_6_ and PLGA is FDA-approved for human use [28], the KGYY_6_ peptide may be viable as a PLGA-formulated treatment option in MS. For GA, it would be necessary to develop a delivery method that does not create a hyper-acidic microenvironment; there are many different types of nano- and microparticle formulations available [32]. As the nano- and microparticle field develops particle types that more readily encapsulate peptides, the loss of peptides in manufacturing may also be lessened. In the future, it will be important to investigate whether even less frequent injections, perhaps as seldom as once every two weeks, can ameliorate disease.

## 5. Conclusions

We found that a slow-release formulation of the MS drug GA, as well as of the CD40-targeting peptide KGYY_6_, vastly improves the efficacy of the drug compared to liquid formulations. In addition, we found that GA efficacy differs from lot to lot while the efficacy of KGYY_6_ remains the same from lot to lot. Our findings may lead to improvements in drug delivery in MS.

## Data Availability

The data sets used and/or analyzed during the current study are available from the corresponding author upon reasonable request. The data are included in Figure S1.

## Supplementary Materials

The following supporting information can be downloaded at: https://www.mdpi.com/article/10.3390/neurolint16060114/s1, Figure S1: Minimal dataset.
