# Supplementary material for: Slow Subcutaneous Release of Glatiramer Acetate or CD40-Targeting Peptide KGYY6 Is More Advantageous in Treating Ongoing Experimental Autoimmune Encephalomyelitis
Source: Neurol Int. 2024 Nov 20;16(6):1540–51. doi: 10.3390/neurolint16060114 (PMC11587089; doi:10.3390/neurolint16060114)
Supplement: Supplementary file 1 [file neurolint-16-00114-s001.zip › neurolint-3318713-supplementary.pdf]

Figure S1. Minimal Dataset.

Detailed EAE induction protocol:

An emulsion of MOG and CFA is created as follows: (It is necessary to make about 1.5-2 times the volume calculated for all mice)

- Fit 2 Luer lock syringes (size depending on the desired final volume of emulsion) to 2 of the 3 ports on a 3-way stopcock with male Luer locks. Place the contraption on ice with one syringe pointing up.
- With the stopcock “off” position to the upward-pointing syringe, pull the plunger out of the syringe and pipet the desired amount of MOG_{35-55} (50 µg per mouse; any source that sells MOG_{35-55}; a stock of 20/mg/ml in PBS) into the syringe. Add an amount of PBS such that the total volume is enough for 50 µl per mouse.
- Carefully fit the plunger back into the syringe and turn the contraption such that the liquid hits the plunger.
- Turn the “off” position to the other syringe then push the liquid in the first one all the way into, but not out of, the stopcock.
- Pull the plunger out of the other syringe and place it on ice with that syringe pointing up. Pipet the desired amount of H37RA (complete Freund’s adjuvant (CFA); a 10 mg/ml stock of H37RA in mineral oil; 75 µg H37RA per mouse), then add an amount of mineral oil (cat# F5506; Sigma) such that the total volume is enough for 50 µl per mouse.
- Carefully fit the plunger back into the syringe and turn the contraption such that the liquid hits the plunger.
- Turn the “off” position to the first syringe then push the liquid in the second one all the way into, but not out of, the stopcock.
- Turn the “off” position to the third stopcock position (without a fitted syringe).
- Push the liquids from one syringe to the other 100 times then place the contraption on ice to chill for a few minutes.
- Repeat the pushing of the liquid for a total of 5 x 100 times, resting on ice after every 100 pushes. The emulsion should be thick and white.

Pertussis toxin (PT) is prepared as follows:

- Pipet the desired amount of PT (200 ng per mouse; cat# P7208; Sigma) into a vial and add an amount of PBS such that the total volume is enough for 100 µl per mouse.
- Draw up the solution into 1 ml syringe(s) fitted with $^{5}/_{8}$-inch long 25-gauge needles and push the solution to the tip of the needle, ensuring that any bubbles are eliminated.

Mice are injected as follows:

- Take the syringe that does not have the emulsion in it off from the contraption. To the same port, fit a 1 ml Luer lock syringe then push a small amount of emulsion (about 100 µl) into it.

- Take the 1 ml syringe off from the stopcock then pull the plunger to bring the emulsion into the syringe. Slowly push the emulsion to the very tip of the syringe allowing a small bead to come out.
- Fit the 1 ml syringe back onto the stopcock then fill it to 0.6 ml.
- Fit a $^{5}/_{8}$-inch long 25-gauge needle onto the 1 ml syringe and carefully push the emulsion to the top of the needle. This syringe is now ready to inject 5 mice with 100 µl emulsion each.
- Restrain a mouse by grabbing the scruff of the neck. Into the scruff, subcutaneously inject 100 µl emulsion then place the mouse in a separate cage. Continue with the other 4 mice.
- When all five mice have received emulsion, restrain them again and inject 100 µl PT intraperitoneally. Place the mice back in their home cage(s).
- One or two days after the initial injections, give the mice an intraperitoneal booster of PT the same way and same amount as before.

NOTE i: Weigh the mice on the day of disease induction to have a baseline weight. Weigh the mice occasionally during the first week but then daily once disease symptoms begin.

NOTE ii: Score the mice daily, especially once disease symptoms begin. Symptoms generally begin to appear 11 days post-induction.

EAE disease scoring:

(Scoring should be performed daily.)

0 – No abnormalities.

0.5 – Clutching hind limbs. (Usually, when mice are picked up by the tail they spread their hind limbs straight out.) Hesitation and/or confusion. (Usually if a mouse is put down on the cage lid it scurries around immediately.)

1 – Limp tail <u>or</u> weak hind limbs and/or wobbly gait.

1.5 – Limp tail <u>or</u> weak hind limbs and/or wobbly gait <u>and</u> clutching hind limbs/hesitation/confusion.

2 – Limp tail <u>and</u> weak hind limbs resulting in wobbly gait. Mouse supports and propels itself using hind limbs. If placed on back, the mouse cannot right itself immediately.

2.5 – Limp tail <u>and</u> weak hind limbs resulting in wobbly gait. Mouse cannot support and properly propel itself using hind limbs but the paws are moving.

3 – Limp tail <u>and</u> one weak hind limb, while the other is completely paralyzed. Mouse still uses the weak hind limb to propel itself somewhat. Mainly the front paws are used for locomotion.

3.5 – Limp tail <u>and</u> one weak hind limb, while the other is completely paralyzed. Mouse does not use the weak limb, which may paddle but is almost at paralysis.

4 – Limp tail and complete hind limb paralysis. (Often the hind limbs are limp and just dragging as the mouse moves. However, occasionally the mice at stage 4 demonstrate spasticity. That is, the

paralyzed limbs are locked in a very tense muscle spasm that will not relax. Note this if present. This is one of the most common symptoms in human MS.)

4.5 – Complete hind limb paralysis and weak fore limb(s). Mouse cannot move around in the cage much. All mice with a score of 4.5 must be euthanized.

5 – Complete paralysis of hind quarter and weak or paralyzed fore limb(s). (There are some more unusual symptoms that we score as a 5 as well. Those are either when a mouse spins uncontrollably or when a mouse is laying on the side unable to propel itself even though it is able to move its paws.) All mice with

a score of 5 must be euthanized.

- Generally, the mice lose weight when at stage 2 or above but still feed themselves up to stage 4 when provided moist chow on the cage floor.
- At stage 4.5 and 5, the mice are usually not able to feed and drink. If they reach a score of 4.5 or 5, or lose more than 15% of their body weight that does not regain within 2-3 days, they must be euthanized immediately.
- When the mice reach a level 3.5, moist food must be provided on the cage floor daily.
- Occasionally, mice display confusion and tend to run around the edges of the cage, leaning on the cage wall. Note these and other unusual symptoms on the scoring sheet.

NOTE: It is important that the same person scores the mice daily as much as possible as there may be subjectivity in the interpretation of the symptoms. For example, at stage 2 the mouse can appear more or less weak while not yet being at a score of 2.5. Therefore, it is important that the same judgment be applied each day. A second person could be scoring daily as well and then the scores could be averaged.

Raw data; Figure 1:

Figure 1A:

Control										
**Day**	**871 R**	**871 L**	**871 LR**	**871 2R**	**871 NC**	**872 R**	**872 NC**	**917 R**	**918 R**	**919 R**
1	0	0	0	0	0	0	0	0	0	0
2	0	0	0	0	0	0	0	0	0	0
3	0	0	0	0	0	0	0	0	0	0
4	0	0	0	0	0	0	0	0	0	0
5	0	0	0	0	0	0	0	0	0	0
6	0	0.5	0	0	0	0	0	0	0	0
7	0	0	0.5	0	0	0.5	0	0	0	0
8	0	0	0	0.5	0	0	0	0	0.5	0
9	0	0.5	0	0	0	0	0	0	0	0
10										
11										
12	0	0.5	3.5	0.5	0	0.5	1.5	2	0	0
13	0.5	3.5	4	0	0	1	2	2.5	0.5	0.5
14	2	4	4	0.5	0.5	1.5	2.5	4	2	0
15	3	4	4	1	0	2	4	4	2	0

16	4	4	5	1.5	0	2	4	5	4	2
17	5	5	5	3.5	0.5	3.5	4	5	4	3.5
18	5	5	5	3.5	0	2.5	4	5	4	4
19	5	5	5	4	0	2	4	5	4	5
20	5	5	5	4	0.5	2	4	5	4	5
21	5	5	5	4	0.5	2	3.5	5	4	5

**Contol**										
**Day**	**490 R**	**490 NC**	**491 2R**	**492 LR**	**478 L**	**479 R**	**479 L**	**479 LR**	**479 2R**	**479 NC**
1	0	0	0	0	0	0	0	0	0	0
2										
3										
4										
5	0	0	0	0	0	0	0	0	0	0
6	0	0	0	0	0	0	0	0	0	0
7	0	0	0	0	0	0	0	0	0	0
8	0	0	0	0	0	0	0	0	0	0
9										
10										
11	0.5	0	0	0	2	0.5	2	0.5	0	0
12	0.5	0	0.5	2	4	2	2.5	2	2	0
13	0	0	2	2.5	4	3	3.5	2	2.5	0
14	0.5	1.5	2.5	3.5	4	4	3.5	3	3.5	2
15	1	2	3	4	4	4	4	3.5	4	2.5
16										
17										
18	2.5	2.5	3	2	2.5	4	2	2.5	2	4
19	4	2	2.5	2	2.5	3	2	4	2	4
20	4	2	2.5	2	2.5	2.5	2	2	2	3
21	4	2	2.5	2	2	2	2	2	2	3

**Control**										
**Day**	**119 R**	**119 L**	**119 LR**	**119 2R**	**119 NC**	**120 R**	**120 L**	**120 LR**	**120 2R**	**120 NC**
1	0	0	0	0	0	0	0	0	0	0
2										
3										
4										
5	0	0.5	0	0	0	0.5	0	0.5	0	0
6	0	0.5	0	0	0	0.5	0	0	0	0
7	0	0.5	0.5	0	0	0	0	0	0	0
8	0	0.5	0	0	0	0	0	0	0	0.5
9	0	0.5	0	0	0	0	1	0	0	0.5
10	0	0.5	0	0	0	0	0.5	0	0	0
11										
12	0.5	0.5	0	2	1	0	2	2	0	0.5
13	1.5	1	0.5	2	2	0.5	2.5	2.5	0.5	0
14	1.5	1	0.5	4	3	0.5	4	2.5	0	0.5
15	2	1	1.5	4	4.5	0.5	4.5	3.5	0	1
16	3	1	1.5	2.5	4.5	1.5	5	2.5	0.5	1
17	4	0.5	2	2	5	2	5	2	0.5	0

18	4	0.5	2	2	5	3	5	2	0	1
19	3	1	2	2	5	4.5	5	2	0.5	0.5
20	2.5	2	2	2	5	5	5	2	0	0
21	2	2	2	2	5	5	5	2	0	0

Control										
**Day**	**725 R**	**725 L**	**725 LR**	**725 2R**	**725 NC**	**726 R**	**726 L**	**726 LR**	**726 2R**	**726 NC**
1	0	0	0	0	0	0	0	0	0	0
2										
3										
4										
5	0	0	0	0	0	0	0.5	0	0.5	0
6	0	0	0	0	0	0	0	0	0	0
7	0	0	0	0	0	0	0	0	0	0
8	0	0	0	0	0	0	0	0	0	0
9										
10										
11	0	0	0	0	0	0	0	0	0	0
12	0.5	0	0.5	0	0	0	0	0	0	0
13	0	1	2	0	0.5	0	0	0	0	0
14	0	2	2.5	0.5	0.5	0	0	0	0	0
15	0	3	4	0.5	0	0	0	1	0	0
16										
17	0	4	4	2	0	0	0	2	1	0
18	0.5	4	2.5	3	0	0	0	2	1	0
19	0	4	2.5	3	0.5	1	0	2	2	1
20	0	3	2.5	4	1	0.5	1	2	3	1
21	0	2.5	2	2.5	1.5	1	0.5	2	3	0.5

GA liquid; 0.6 mg/kg; 3x per week; Lot# 1										
**Day**	**868 R**	**868 L**	**868 LR**	**868 2R**	**868 NC**	**872 2R**	**917 2R**	**917 NC**	**918 NC**	**919 NC**
1	0	0	0	0	0	0	0	0	0	0
2	0	0	0	0	0	0	0	0	0	0
3	0	0	0	0	0	0	0	0	0	0
4	0	0	0	0	0	0	0	0	0	0
5	0	0	0	0	0	0	0	0	0	0
6	0	0	0	0	0	0	0	0	0	0
7	0	0	0	0	0	0	0	0	0	0
8	0	0	0	0	0	0	0.5	0	0	0
9	0	0	0	0	0	0	0	0	0	0
10										
11										
12	1.5	0	0	0	0	0	1.5	0	0	0
13	2	0	0	0	0	0	2.5	0	0	1.5
14	3.5	0	0	0.5	2	0.5	3.5	0	1.5	3.5
15	3.5	1.5	1.5	0.5	2	0	4	0	2	4
16	4	2.5	2	0.5	2	1.5	4	0	4	5
17	4	4	2.5	0.5	3.5	2	5	0	4	5
18	5	5	3.5	0.5	3.5	2.5	5	0	5	5
19	5	5	3.5	2	3.5	4	5	0	5	5
20	5	5	2.5	2	3.5	4	5	0	5	5
21	5	5	2	2.5	2.5	4	5	0	5	5

GA liquid; 100 mg/kg; 5x per week; Lot# 1										
Day	490 L	491 R	491 NC	492 2R	478 LR	480 R	480 L	480 LR	480 2R	480 NC
1	0	0	0	0	0	0	0	0	0	0
2										
3										
4										
5	0	0	0	0	0	0	0	0	0	0
6	0	0	0	0	0	0	0	0	0	0
7	0	0	0	0	0	0	0	0	0	0
8	0	0	0	0	0	0	0	0	0	0
9										
10										
11	0	0	0	0	0	0	0	0	1.5	0
12	0	0.5	0	0	0	0	0	0	4	0
13	0	2	0	0.5	0	0	0	0	4	0
14	1.5	3.5	2	0	0	0	0	0	4	0
15	2	4	3	0.5	2	0	0	0.5	4	1.5
16										
17										
18	4	4	4	1	2	2	2	4	4	4
19	4	3.5	2	1	2	2	2	2.5	4	4
20	2.5	2.5	2	1.5	1.5	2.5	2	2.5	4	4
21	2	2	2	1.5	1	4	2	2.5	4	2.5

KGYY6 liquid; 4 mg/kg; 3x per week										
Day	490 LR	491 L	492 R	492 NC	478 2R	481 R	481 L	481 LR	481 2R	481 NC
1	0	0	0	0	0	0	0	0	0	0
2										
3										
4										
5	0	0	0	0	0	0	0	0	0	0
6	0	0	0	0	0	0	0	0	0	0
7	0	0	0	0	0	0	0	0	0	0
8	0	0	0	0	0	0	0	0	0	0
9										
10										
11	0	0	0	0	0	0	0	0	0	0
12	0	2	0	0	1	0	0	0	0	0
13	0	2	0	2	1.5	0	0	1	0	0
14	0	3.5	0.5	2	3	0	0.5	2	1.5	0
15	0	2.5	2	3	4	0	2	4	2	0.5
16										
17										
18	2	2.5	4	2	3	0	4	2.5	4	2
19	2	2	2.5	2	2	0	3.5	2.5	4	2
20	2	2	2	2	2	0	2.5	2.5	4	2
21	2	2	2	2	2	0	2	2.5	3	2

KGYY6 liquid; 8 mg/kg; 3x per week										
Day	490 2R	491 LR	492 L	478 R	478 NC	482 R	482 L	482 LR	482 2R	482 NC
1	0	0	0	0	0	0	0	0	0	0
2										
3										
4										
5	0.5	0	0	0	0	0	0	0	0	0
6	0	0	0	0	0	0	0	0	0	0
7	0	0	0	0	0	0	0	0	0	0
8	0	0	0	0	0	0	0	0	0	0
9										
10										
11	0	0	0	0	0	0	0	2	0	0
12	0	0	0	0	0	0	0	4	0	0
13	0	1	2	0	0	0	0	4	0	0.5
14	0	2	2	0	0	2	1	4	0	2
15	0	3	2.5	1	0	2.5	2	4	0	2.5
16										
17										
18	0	3	2	3.5	1.5	3	2	2.5	0	2
19	0	2	2	3	2	2	2	2.5	0	2
20	0	2	2	2.5	2.5	2	2	2.5	0	2
21	0	2	2	2	3	2	2	2	0	2

KGYY6 liquid; 8 mg/kg; 3x per week										
Day	127 R	127 L	127 LR	127 2R	127 NC	128 R	128 L	128 L	128 2R	128 NC
1	0	0	0	0	0	0	0	0	0	0
2										
3										
4										
5	0.5	0	0	0	0	0	0	0	0	0
6	0	0	0	0	0	0	0	0	0	0
7	0.5	0	0	0	0	0	0	0	0	0
8	0	0	0	0	0	0	0	0	0	0
9	0	0	0	0	0	0	0	0	0	0
10	0	0	0	0	0	0	0	0	0	0.5
11										
12	0	1	2	0	0	0	0	0	0	0
13	1	2	2.5	1	1	0	0	0	0	0
14	1	2.5	4	1.5	2.5	0	0	0	1	1
15	2	2.5	3.5	1	3	0	0	1	2	2
16	3.5	3.5	2.5	2	3.5	0	0	1.5	4	4
17	4	3.5	2.5	1.5	3	0	1	2	3.5	4
18	3	2.5	2.5	2	2.5	0	1	3.5	4.5	4
19	2.5	2.5	2.5	2	2	0	1	4	5	4
20	2	2.5	4.5	1	2	0	1	4.5	5	4
21	2	2	5	1	2	0	0.5	5	5	2

KGYY6 liquid; 8 mg/kg; 3x per week										
Day	723 R	723 L	723 LR	723 2R	723 NC	724 R	724 L	724 LR	724 2R	724 NC
1	0	0	0	0	0	0	0	0	0	0
2										
3										
4										
5	0	0	0.5	0	0	0	0	0	0.5	0
6	0	0	0	0	0	0	0	0	0	0
7	0	0	0	0	0	0	0	0	0	0
8	0	0	0	0	0	0	0	0	0	0
9										
10										
11	0	0	0	0	0	0	0	0	0	0
12	0	0	0	0	0	0	0	0	0	0
13	0	0	0	0	0	0	0	0	0	0
14	0	0	2	1	0	0	0	0	0	0.5
15	0	0	2.5	1	0	0	0	0	0	2
16										
17	0.5	2	4	2	2	0	0	0	0	2
18	1	3	4	1.5	2.5	0	0	0	0	3
19	1.5	3	4	1.5	4	0	0	0.5	0	2
20	2	4	2	1.5	4	0	0.5	0.5	0	2
21	2	4	2	1	4	0	1	0	0	1.5

GA liquid; 100 mg/kg; 5x per week; Lot# 2										
Day	121 R	121 L	121 LR	121 2R	121 NC	122 R	122 L	122 LR	122 2R	122 NC
1	0	0	0	0	0	0	0	0	0	0
2										
3										
4										
5	0	0	0	0	0	0	0	0	0.5	0
6	0	0.5	0	0	0	0	0	0.5	0.5	0
7	0	0	0	0	0	0	0	0	0.5	0.5
8	0	0	0.5	0	0	0	0.5	0	0.5	0
9	0	0.5	0	0	0	0.5	0	0	0.5	0.5
10	0	0.5	0	0.5	0	0	0	0	0.5	0.5
11										
12	1	2.5	0	0.5	0	0.5	0	0	0.5	0
13	2	4	0.5	0.5	0.5	1	0	0.5	0.5	0
14	2.5	4.5	0	1	0.5	2	0	1	0.5	0
15	4.5	4.5	0	1	0.5	2.5	0	2	0.5	0.5
16	4	5	0.5	2	0	3	0	2	0.5	1
17	4.5	5	1.5	3	0.5	4.5	0.5	4	0.5	1
18	5	5	2	4	0	5	0	4.5	0.5	1.5
19	5	5	2	4	0	5	0	5	0.5	1.5
20	5	5	2	3	0.5	5	0	5	0.5	1.5
21	5	5	2	3	0.5	5	0	5	1.5	2

Figure 1B:

Control										
**Day**	**871 R**	**871 L**	**871 LR**	**871 2R**	**871 NC**	**872 R**	**872 NC**	**917 R**	**918 R**	**919 R**
1	2.9	1.4	3.5	2.4	1.4	1.4	-0.5	2.5	1.2	-0.7
2										
3										
4										
5										
6	5.3	11.2	12.9	10.4	3.8	4.1	5.9	6	5.4	6.6
7	5.3	13.1	13.4	11.3	3.8	4.7	6.3	6.4	4.3	9
8	7.7	15	17.3	13.7	5.2	5.4	10.2	7.5	5.4	9.7
9	8.2	14.5	19.8	14.2	5.7	5.4	9.8	7.5	4.7	11.1
10										
11										
12	6.7	4.7	-9.4	17.5	8.1	7.4	-1.5	-11.4	5	11.1
13	5.8	-3.7	-13.9	13.2	5.2	6.8	-11.7	-15.7	1.9	9
14	-2.9	-10.7	-15.3	15.1	8.6	5.4	-16.1	-16.4	-8.1	13.5
15	-10.6	-12.1	-14.9	13.7	9.5	-3.4	-16.1	-14.9	-16.3	13.2
16	-17.3	-14	-16.3	10.4	10.5	-7.4	-15.6	-15.7	-18.6	10.8
17										
18	-17.3	-14	-16.3	-5.7	8.1	-6.8	-18.5	-15.7	-17.4	-8.3
19	-17.3	-14	-16.3	-8	9	-7.4	-21	-15.7	-19.8	-8.3
20	-17.3	-14	-16.3	-7.5	11.4	-5.4	-21	-15.7	-17.1	-8.3
21	-17.3	-14	-16.3	-2.8	2.4	-1.4	-17.1	-15.7	-16.7	-8.3

Control										
**Day**	**490 R**	**490 NC**	**491 2R**	**492 LR**	**478 L**	**479 R**	**479 L**	**479 LR**	**479 2R**	**479 NC**
1	0	0.8	0.4	-0.4	1.2	2.5	3.3	-0.6	1.6	3.4
2										
3										
4										
5	2.2	4.2	5.3	0	9	7.1	6.5	7.6	5.8	7.8
6	4	4.6	7.3	0.4	12	8.6	8.7	8.8	7.4	8.9
7	6.2	7.6	7.3	1.6	13.3	13.2	12	11.1	7.9	10.6
8	4.8	5.1	8.2	2.8	13.9	15.2	14.7	11.7	11.6	10.1
9										
10										
11	4	4.6	10.6	1.6	9	10.2	1.1	5.3	10.6	10.1
12	8.8	3.8	6.5	-7.1	-4.2	-4.6	-9.2	-6.4	-3.2	12.8
13	6.6	2.1	-4.1	-15	-9.6	-9.6	-13.6	-5.8	-11.6	7.3
14	7	-0.8	-13.1	-19.4	-7.2	-10.2	-10.9	-4.1	-10.6	-2.2
15	6.2	-6.3	-13.1	-18.6	-7.2	-10.7	-9.8	-4.1	-13.2	-10.1
16										
17										
18	-12.8	-9.7	-16.7	-15	-3	-13.2	-3.3	-11.7	-6.3	-16.2
19	-19.4	-7.6	-14.7	-13.8	-2.4	-10.2	0.5	-12.3	-2.6	-14.5
20	-19.4	-5.9	-12.2	-13.4	0.6	-8.6	2.2	-9.4	-0.5	-14.5
21	-20.7	-4.2	-12.2	-11.5	1.8	-7.6	4.3	-5.8	0.5	-13.4

Control										
Day	119 R	119 L	119 LR	119 2R	119 NC	120 R	120 L	120 LR	120 2R	120 NC
1	0	0	0	0	0	0	0	0	0	0
2										
3										
4										
5	4.8	-0.6	4.2	5.9	3	5.6	5.3	7.5	9	5.2
6	4.8	-4.4	7	5.4	4.5	6.9	5.9	9	10.1	5.2
7	6.2	-5.6	7	5.4	3.5	7.9	6.9	9.5	11.2	7.6
8	7.2	-6.3	8.9	6.9	4.5	11.6	7.4	10.1	13.8	8.6
9	6.7	-6.9	9.4	5.9	5	8.8	7.4	11.6	14.4	8.6
10	8.6	0	8.9	5.9	7	10.6	9.6	13.6	13.8	6.2
11										
12	5.7	1.3	9.4	-8.9	-3.5	7.4	-4.3	-2.5	9.6	4.3
13	-4.3	2.5	7	-13.9	-9.5	7.9	-12.2	-6.5	12.8	7.1
14	-12.4	1.3	4.7	-10.9	-17	6.9	-17.6	-8.5	14.9	7.6
15	-16.3	16.3	9.4	-11.4	-17	6.5	-26.6	-7	16	8.1
16	-16.3	18.8	6.6	-8.9	-18	0.9	-26.6	-6	15.4	7.6
17	-15.3	22.5	2.3	-7.9	-18	-2.3	-26.6	-5	14.9	7.1
18	-16.7	23.8	0.9	-5	-18	-6.9	-26.6	-3.5	12.8	8.6
19	-15.8	21.9	-0.5	-2	-18	-9.3	-26.6	-3	12.8	14.8
20	-12.4	13.8	1.4	-2	-18	-9.3	-26.6	-2.5	14.4	11
21	-10.5	10	1.4	-3	-18	-9.3	-26.6	-1.5	14.9	11.9

Control										
Day	725 R	725 L	725 LR	725 2R	725 NC	726 R	726 L	726 LR	726 2R	726 NC
1	0	0	0	0	0	0	0	0	0	0
2										
3										
4										
5	-2.2	1.3	6.3	-2.2	4.8	-0.4	3.1	-2.7	7.3	10.5
6	-0.9	0	7.3	-3.9	1.4	0.4	0.4	-4.6	8.3	7.4
7	-0.4	0	7.8	-3	3.8	-1.3	0	-3.2	8.3	8.3
8	0.4	1.3	10.7	-1.7	2.4	-0.4	4.4	-2.7	10.7	7
9										
10										
11	-3	0	11.7	-4.3	2.9	-5	3.1	-2.3	8.3	3.9
12	-3	2.2	9.7	-1.7	3.3	-4.2	2.7	-0.9	8.7	7.9
13	-5.2	-2.2	-2.9	-3.5	2.4	-5	5.3	-0.5	8.3	7
14	-3.9	-7.6	-5.3	-2.2	6.7	-5	2.7	-0.5	14.1	5.7
15	-0.9	-14.3	-12.1	-4.8	5.3	-1.3	4	3.7	11.7	5.7
16										
17	3.9	-19.7	-9.2	-15.2	6.7	-0.8	4.9	0.5	9.2	4.4
18	3.5	-20.2	-8.3	-17.8	5.7	-2.5	3.1	0.5	8.7	5.2
19	5.2	-19.3	-7.3	-17	5.7	-0.8	7.6	0.9	6.8	8.3
20	5.6	-13	-5.3	-14.3	0	-0.8	6.7	0	1	9.6
21	5.6	-13.5	-2.4	-14.8	-5.7	-2.9	4	0.9	-1.9	9.2

GA liquid; 0.6 mg/kg; 3x per week; Lot# 1										
Day	868 R	868 L	868 LR	868 2R	868 NC	872 2R	917 2R	917 NC	918 NC	919 NC
1	1.4	3.8	5.3	5.9	3.1	1.4	2.1	-0.4	3.9	1.3
2										
3										
4										
5										
6	10.8	9.5	16.3	14.4	9.9	7.4	5.6	4.3	1.2	1.3
7	11.3	12.4	14.4	15.4	12	7.4	6.9	4.3	1.2	1.7
8	12.3	13.3	12	17	14.1	9.4	5.9	6.8	1.2	0.4
9	13.7	17.1	10.6	19.1	14.1	11.9	5.9	6.8	1.6	0
10										
11										
12	0	17.6	13.9	17	11.5	11.4	6.6	5	4.3	-1.7
13	-9.4	18.1	14.9	18.6	3.1	10.4	9	-2.5	4.7	-11.3
14	-10.4	17.6	6.3	18.1	-7.3	13.9	8	-10.4	1.6	-18.3
15	-10.8	9.5	-3.8	19.1	-15.1	8.9	6.3	-15.8	-3.1	-21.7
16	-9.9	1.9	-11.1	20.7	-19.8	3.5	8	-16.9	-10.9	-23.9
17										
18	-10.8	-10	-13.5	15.4	-14.6	-10.9	8.3	-16.9	-17.4	-23.9
19	-10.8	-10	-12	4.8	-12.5	-13.9	6.3	-16.9	-17.4	-23.9
20	-10.8	-10	-12.5	5.9	-12	-13.4	4.9	-16.9	-17.4	-23.9
21	-10.8	-10	-10.1	2.1	-9.9	-12.4	6.3	-16.9	-17.4	-23.9

GA liquid; 100 mg/kg; 5x per week; Lot# 1										
Day	490 L	491 R	491 NC	492 2R	478 LR	480 R	480 L	480 LR	480 2R	480 NC
1	2.9	3.8	5.7	1.3	-3.7	1	2.1	2.2	5.8	-1
2										
3										
4										
5	5.5	6.8	8.1	3.4	5.8	6	8.4	5.4	9.5	6.8
6	6.3	7.6	8.6	4.7	5.3	9.5	10.5	6	11.6	8.3
7	6.3	8.3	9.1	5.5	6.3	12.5	13.6	7.6	14.8	9.9
8	5.5	9.1	10.5	6.4	8.4	12.5	14.7	8.7	18	8.9
9										
10										
11	8	9.8	11.5	5.1	15.3	11.5	19.4	11.4	4.2	10.4
12	8.4	8	11	7.6	13.2	11.5	20.4	12.5	-3.2	9.9
13	7.1	2.7	9.6	8.1	12.1	15.5	22	10.3	-13.2	10.9
14	0.8	-3.8	7.2	9.3	12.1	16.5	19.4	10.3	-13.2	8.9
15	-7.1	-9.1	-2.4	8.5	10.5	16	13.1	-0.5	-12.7	0
16										
17										
18	-11.8	-10.2	-4.3	0.4	7.4	2	4.7	-11.4	-12.7	-15.6
19	-10.9	-9.8	-3.3	2.1	8.4	-3	6.3	-12.5	-12.7	-13
20	-10.9	-11.7	-0.5	3.8	10	-4	6.3	-10.9	-10.6	-14.6
21	-10.1	-10.2	0	4.2	11.1	-5.5	8.4	-13.6	-11.1	-17.7

KGYY6 liquid; 4 mg/kg; 3x per week										
**Day**	**490 LR**	**491 L**	**492 R**	**492 NC**	**478 2R**	**481 R**	**481 L**	**481 LR**	**481 2R**	**481 NC**
1	1.6	3.4	1.4	0.4	-2.7	3.9	5	3.9	-7.3	5.4
2										
3										
4										
5	6.3	7.6	4.8	4.5	3.3	6.7	10	7.7	-0.5	9.7
6	6.3	7.1	4.3	4.9	6.5	9.6	13.3	8.8	2.6	11.4
7	6.7	9.7	5.3	5.7	8.7	10.7	14.4	11	6.3	14.1
8	6	7.6	5.3	5.3	10.3	12.4	20	12.2	8.4	12.4
9										
10										
11	9.5	8	5.8	5.3	9.2	14.6	17.8	12.7	13.1	14.1
12	13.5	-4.2	7.7	2.8	-2.7	15.2	17.8	6.1	9.9	16.2
13	9.5	-12.2	7.7	-3.6	-8.7	15.2	13.9	-5	8.9	15.1
14	9.9	-8.8	6.3	-10.9	-12.5	19.1	6.1	-11	1.6	13
15	11.1	-7.6	-3.8	-15.8	-15.2	20.8	-3.3	-11.6	-9.9	0.5
16										
17										
18	0.4	-10.5	-12.5	-8.1	-9.8	21.9	-5.6	-13.8	-14.1	-3.8
19	-1.6	-9.2	-11.1	-6.1	-9.2	23.6	-5.6	-12.2	-16.8	-4.9
20	-1.2	-8	-9.1	-4.9	-6.5	26.4	-0.6	-13.3	-15.2	-4.3
21	0.8	-7.1	-7.2	-2.8	-3.8	28.1	-2.8	-12.7	-16.2	-5.4

KGYY6 liquid; 8 mg/kg; 3x per week										
**Day**	**490 2R**	**491 LR**	**492 L**	**478 R**	**478 NC**	**482 R**	**482 L**	**482 LR**	**482 2R**	**482 NC**
1	2.8	1.8	4.1	0.5	-2.9	2.2	0	1	3.3	-1.2
2										
3										
4										
5	5	5.5	6.4	7.7	6.4	10.8	6.1	7.7	10.4	7.9
6	4.6	5	5.9	8.8	6.4	13.4	7.3	9.7	11.5	7.9
7	5.5	5.9	6.4	10.8	9.9	15.1	10.1	13.8	13.7	8.5
8	6	5.9	8.2	12.4	8.8	16.7	12.8	13.3	14.2	11.6
9										
10										
11	8.7	10.5	7.7	11.9	13.5	14.5	10.1	1.5	12	12.8
12	8.3	9.5	-3.2	10.3	13.5	16.7	13.4	-8.7	12	11
13	6.9	5.5	-10.9	1.5	15.8	13.4	5	-12.2	17.5	-0.6
14	7.8	-3.6	-8.6	-9.3	17.5	4.3	-7.3	-13.3	19.1	-8.5
15	8.3	-9.1	-12.7	-14.4	16.4	-3.8	-11.7	-14.3	16.4	-12.2
16										
17										
18	11.5	-13.6	-11.4	-12.4	11.7	-1.6	-8.4	-12.2	16.4	-15.2
19	15.6	-11.8	-9.5	-11.9	7	1.6	-6.1	-12.2	18.6	-11
20	17	-9.1	-7.7	-10.8	1.8	4.8	-2.2	-8.2	19.1	-7.9
21	17	-5.5	-4.5	-12.4	1.2	4.3	0	-7.7	18.6	-4.9

KGYY6 liquid; 8 mg/kg; 3x per week										
Day	125 R	125 L	125 LR	125 2R	125 NC	126 R	126 L	126 LR	126 2R	126 NC
1	0	0	0	0	0	0	0	0	0	0
2										
3										
4										
5	3.3	5.3	7	1.7	7.1	10.2	11	4.3	7.7	4.6
6	1	7.7	5.5	1.3	7.7	8.7	12	2.8	3.3	4.1
7	2.9	10.5	8.5	3.5	11.7	7.3	14.4	1.4	7.7	5.1
8	4.8	11.5	11.6	6.1	15.8	5.3	18.7	5.7	8.2	7.8
9	4.3	10	10.6	5.6	12.2	4.4	15.3	5.7	8.8	6.5
10	5.7	11.5	13.1	7.4	15.8	3.9	14.8	6.6	7.1	7.4
11										
12	-14.8	-1.9	-1.5	6.9	11.7	6.3	6.2	3.3	6.6	2.8
13	-18.7	-7.2	-4.5	8.7	12.2	7.3	-0.5	3.8	8.2	8.8
14	-21.1	-12.9	-10.1	10	11.2	7.3	-4.8	5.7	8.8	11.1
15	-20.1	-12.9	-13.6	10	12.2	8.3	-4.3	5.7	7.7	8.8
16	-18.7	-12	-10.6	12.1	11.2	9.2	-2.9	6.2	7.7	9.2
17	-18.2	-12	-10.1	9.1	10.2	11.2	-0.5	10	9.3	10.6
18	-15.8	-11	-7	9.5	11.7	11.2	6.2	8.5	7.1	10.6
19	-13.4	-9.1	-6.5	10.4	11.7	11.2	7.2	8.1	5.5	12.9
20	-12.9	-7.7	-6	10.4	10.2	12.6	8.6	8.1	4.4	12
21	-12.9	-6.2	-4.5	10.8	9.7	10.2	8.6	6.6	4.9	10.1

KGYY6 liquid; 8 mg/kg; 3x per week										
Day	723 R	723 L	723 LR	723 2R	723 NC	724 R	724 L	724 LR	724 2R	724 NC
1	0	0	0	0	0	0	0	0	0	0
2										
3										
4										
5	-0.9	0.4	1.9	4	-3.2	4.1	2.4	-2.8	3.7	4.7
6	0.5	0.9	2.9	2.5	-5	4.1	1.6	-2.8	2.8	6
7	2.3	0.4	2.9	4.5	-2.3	5.5	2.8	-5	3.7	6.5
8	2.3	3.6	6.7	6	-0.9	4.1	4	-4.1	4.7	7.4
9										
10										
11	2.7	-0.4	7.2	3	0.5	-0.5	-0.4	-4.6	0.5	4.2
12	0.5	2.7	7.2	5.5	-3.2	0.5	2.4	-3.2	1.9	4.7
13	-1.4	-0.9	2.4	5	-2.7	1.8	-0.4	-0.5	4.2	6.5
14	0.5	-0.4	-4.8	1.5	-2.3	2.3	2	-1.4	4.2	5.1
15	1.8	-3.6	-14.4	-3	0	1.8	2.4	-1.8	2.3	-0.9
16										
17	2.7	-9.9	-15.4	-1	-12.6	0.9	6	0	3.7	-9.8
18	-0.9	-16.6	-13.9	-1.5	-20.3	4.6	7.2	-0.9	6.5	-12.1
19	-2.3	-18.8	-12.5	0	-26.1	5	5.6	0.5	7.5	-7.4
20	-4.6	-18.4	-12	-1	-25.2	-1.4	5.2	3.2	4.2	-2.8
21	-4.1	-17.5	-11.1	0	-26.6	4.6	3.2	3.7	4.7	-4.7

GA Liquid; 100 mg/kg; 5x per week; Lot# 2										
Day	121 R	121 L	121 LR	121 2R	121 NC	122 R	122 L	122 LR	122 2R	122 NC
1	0	0	0	0	0	0	0	0	0	0
2										
3										
4										
5	9.5	4.9	8.7	4.6	-0.9	8	-0.9	4.6	6.3	4.2
6	8.5	7.6	8.2	6	1.3	6.5	0	5.7	4.4	3.8
7	10.5	9.8	8.7	9.6	3.1	8	2.6	2.9	5.4	7
8	12.5	11.6	11.7	11.5	5.7	10.1	6.6	13.1	8.8	10.8
9	13.5	6.7	12.2	10.6	4.8	8	7.4	14.9	8.3	8
10	13.5	7.6	15.3	10.6	5.3	10.6	5.7	13.7	6.8	7.5
11										
12	9	-12.1	10.7	5.5	3.1	5	5.2	12	5.9	6.6
13	0	-15.2	17.9	12.4	12.3	-2	7.4	12.6	7.8	9.4
14	-8	-19.2	17.3	11	8.4	-10.1	5.2	14.3	7.8	8.9
15	-12.5	-19.6	16.3	11	9.7	-13.6	4.8	7.4	6.8	6.6
16	-14.5	-19.6	14.8	7.8	9.7	-16.6	7	0	9.8	6.6
17	-20.5	-19.6	12.2	0.5	9.3	-19.1	6.6	-8.6	7.8	11.7
18	-20.5	-19.6	9.7	-7.8	11.9	-19.1	7.4	-12.6	10.2	10.8
19	-20.5	-19.6	8.2	-11	10.1	-19.1	10.9	-12.6	11.2	9.4
20	-20.5	-19.6	5.6	-8.7	9.7	-19.1	9.6	-12.6	11.7	9.9
21	-20.5	-19.6	2	-11	8.8	-19.1	8.3	-12.6	9.3	5.6

Raw data; Figure 2:

Figure 2C:

	Absorbance		
Day	Exp.1	Exp.2	Exp.3
0	0.286	0.309	0.330
1	0.416	0.392	0.438
2	0.456	0.475	0.558
6	0.718	0.772	0.731
7	0.899	0.872	0.870
8	0.979	0.978	0.991
9	1.031	0.988	1.063
13	1.102	1.205	1.152
16	1.154	1.168	1.217
20	1.170	1.257	1.180

Raw data; Figure 3:

Figure 3A:

Control										
**Day**	**871 R**	**871 L**	**871 LR**	**871 2R**	**871 NC**	**872 R**	**872 NC**	**917 R**	**918 R**	**919 R**
1	0	0	0	0	0	0	0	0	0	0
2	0	0	0	0	0	0	0	0	0	0
3	0	0	0	0	0	0	0	0	0	0
4	0	0	0	0	0	0	0	0	0	0
5	0	0	0	0	0	0	0	0	0	0
6	0	0.5	0	0	0	0	0	0	0	0
7	0	0	0.5	0	0	0.5	0	0	0	0
8	0	0	0	0.5	0	0	0	0	0.5	0
9	0	0.5	0	0	0	0	0	0	0	0
10										
11										
12	0	0.5	3.5	0.5	0	0.5	1.5	2	0	0
13	0.5	3.5	4	0	0	1	2	2.5	0.5	0.5
14	2	4	4	0.5	0.5	1.5	2.5	4	2	0
15	3	4	4	1	0	2	4	4	2	0
16	4	4	5	1.5	0	2	4	5	4	2
17	5	5	5	3.5	0.5	3.5	4	5	4	3.5
18	5	5	5	3.5	0	2.5	4	5	4	4
19	5	5	5	4	0	2	4	5	4	5
20	5	5	5	4	0.5	2	4	5	4	5
21	5	5	5	4	0.5	2	3.5	5	4	5

	Control										
	**Day**	**490 R**	**490 NC**	**491 2R**	**492 LR**	**478 L**	**479 R**	**479 L**	**479 LR**	**479 2R**	**479 NC**
	1	0	0	0	0	0	0	0	0	0	0
	2										
	3										
	4										
	5	0	0	0	0	0	0	0	0	0	0
	6	0	0	0	0	0	0	0	0	0	0
	7	0	0	0	0	0	0	0	0	0	0
	8	0	0	0	0	0	0	0	0	0	0
	9										
	10										
	11	0.5	0	0	0	2	0.5	2	0.5	0	0
	12	0.5	0	0.5	2	4	2	2.5	2	2	0
	13	0	0	2	2.5	4	3	3.5	2	2.5	0
	14	0.5	1.5	2.5	3.5	4	4	3.5	3	3.5	2
	15	1	2	3	4	4	4	4	3.5	4	2.5
	16										
	17										
	18	2.5	2.5	3	2	2.5	4	2	2.5	2	4
	19	4	2	2.5	2	2.5	3	2	4	2	4
	20	4	2	2.5	2	2.5	2.5	2	2	2	3
	21	4	2	2.5	2	2	2	2	2	2	3

Control										
Day	725 R	725 L	725 LR	725 2R	725 NC	726 R	726 L	726 LR	726 2R	726 NC
1	0	0	0	0	0	0	0	0	0	0
2										
3										
4										
5	0	0	0	0	0	0	0.5	0	0.5	0
6	0	0	0	0	0	0	0	0	0	0
7	0	0	0	0	0	0	0	0	0	0
8	0	0	0	0	0	0	0	0	0	0
9										
10										
11	0	0	0	0	0	0	0	0	0	0
12	0.5	0	0.5	0	0	0	0	0	0	0
13	0	1	2	0	0.5	0	0	0	0	0
14	0	2	2.5	0.5	0.5	0	0	0	0	0
15	0	3	4	0.5	0	0	0	1	0	0
16										
17	0	4	4	2	0	0	0	2	1	0
18	0.5	4	2.5	3	0	0	0	2	1	0
19	0	4	2.5	3	0.5	1	0	2	2	1
20	0	3	2.5	4	1	0.5	1	2	3	1
21	0	2.5	2	2.5	1.5	1	0.5	2	3	0.5

GA PLGA-particles; 4 mg/kg										
Day	869 R	869 L	869 LR	869 2R	869 NC	872 L	917 L	918 L	919 L	919 LR
1	0	0	0	0	0	0	0	0	0	0
2	0	0	0	0	0	0	0	0	0	0
3	0	0	0	0	0	0	0	0	0	0
4	0	0	0	0	0	0	0	0	0	0
5	0	0	0	0	0	0	0	0	0	0
6	0	0	0	0	0	0	0	0	0	0
7	0	0	0	0	0	0	0	0	0	0
8	0	0	0	0	0	0	0	0	0	0
9	0	0	0	0	0	0	0	0	0	0
10										
11	0	0	0	0	0	0	0	0	0	0
12	0	0	0	2	1.5	1.5	0.5	0	0	0
13	0	0	0	2	3	2	0	0	1	1.5
14	0	0	2	2.5	4	3.5	0.5	0.5	2	2.5
15	0	1.5	3.5	2.5	4	4	0.5	2	2.5	4
16	0	2	4	2	4	4	0.5	2	4	4
17	0	3.5	4	2	4	4	0	2.5	4	4
18	0.5	3.5	4	2	4	4	0	3.5	4	3.5
19	2	3.5	4	2	3.5	4	0	2.5	4	2.5
20	2	3.5	3.5	2	3.5	3.5	0	2	3.5	2.5
21	3.5	2.5	3.5	2	2.5	2.5	0	2	2.5	2

GA PLGA-particles; 8 mg/kg; Lot# 1										
**Day**	**987 R**	**987 L**	**987 LR**	**987 2R**	**987 NC**	**018 R**	**018 L**	**018 LR**	**018 2R**	**018 NC**
1	0	0	0	0	0	0	0	0	0	0
2	0	0	0	0	0	0	0	0	0	0
3	0	0	0	0	0	0	0	0	0	0
4	0	0	0	0	0	0	0	0	0	0
5	0	0	0	0	0	0	0	0	0	0
6	0	0	0	0	0	0	0	0	0	0
7	0	0	0	0	0	0	0	0	0	0
8	0	0	0	0	0	0	0	0	0	0
9	0	0	0	0	0	0	0	0	0	0
10										
11	0	0	0	0	0	0	0	0	0	0
12	0	0	0	0	0	0	0	0	0	0
13	0	0	0	0	0	0	0	0	0	0
14	0	0	0	0	0.5	0	0	0	0	0.5
15	0	0	2	0	2	0	0	0	0	0
16	0	0	2	0	3.5	0	0	0	0	0.5
17	0	0	2	0	3.5	0	0	0	0	0.5
18	0	0	2	0	3.5	0	0	0	0	0.5
19	0	0	2.5	0	4	0	0	0	0	0
20	0	0	2.5	0	4	0	0	0	0	0
21	0	0	2.5	0.5	2.5	0	0	0	0	0

GA PLGA-particles; 8 mg/kg; Lot# 2										
**Day**	**719 R**	**719 L**	**719 LR**	**719 2R**	**719 NC**	**720 R**	**720 L**	**720 LR**	**720 2R**	**720 NC**
1	0	0	0	0	0	0	0	0	0	0
2										
3										
4										
5	0	0	0	0	0	0	0	0	0	0
6	0	0	0	0	0	0	0	0	0	0
7	0	0	0	0	0	0	0	0	0	0
8	0	0	0	0	0	0	0	0	0	0
9										
10										
11	0	0	0	0	0	0	0	0	0	0
12	0	0	0	0	0	0	0	0	0	0
13	0	2	0.5	0	0	0	0	1	0	0
14	0	2	2	0	0	0	0	2	0	0
15	0	3.5	2.5	0.5	0	0	0	4	0	0
16										
17	0	4	4	2	0	0	1	4	1	2
18	0	3.5	4	2	0	0	2	4	2	4
19	1	2.5	3	2	0	0	3	4.5	2.5	4.5
20	2	2.5	2.5	2	0	0	3	5	4	5
21	2	2	2.5	2	0	0	2	5	4	5

KGYY$_6$ PLGA particles; 8 mg/kg: lot#1										
**Day**	**807 R**	**807 L**	**807 LR**	**807 2r**	**807 NC**	**810 L**	**810 LR**	**831 NC**	**832 R**	**832 L**
1	0	0	0	0	0	0	0	0	0	0
2	0	0	0	0	0	0	0	0	0	0
3	0	0	0	0	0	0	0	0	0	0
4	0	0	0	0	0	0	0	0	0	0
5	0	0	0	0	0	0	0	0	0	0
6	0	0	0	0	0	0	0	0	0	0
7	0	0	0	0	0	0	0	0.5	0	0
8	0.5	0	0	0	0	0	0	0.5	0.5	0
9	0.5	0	0	0	0	0	0	0.5	0.5	0
10	0.5	0	0	0	0	0	0	0.5	0.5	0
11	0	0	0	0	0	0	0	0	0	0
12	0	0	0	0	0	0	0	0	0	0
13	0	0	0	0	0	0	0	0.5	0.5	0.5
14	0.5	0	0	0	0	0	1	0.5	0.5	0.5
15	0	0	0	0	0	0	2	2	1.5	0
16	0	0	0	0	0	0	2	2	2	0.5
17	0	0	0	0	0	0	2.5	3.5	1.5	0
18	0	0	0	0	0	0	2	4	2	0
19	0	0	0	0	0	0	2	4	2	0.5
20	0	0.5	0	0	0	0	2	4	1.5	0
21	0	0.5	0	0	0	0	2	3.5	2	0.5

KGYY$_6$ PLGA particles; 8 mg/kg: lot#2										
**Day**	**721 R**	**721 L**	**721 LR**	**721 2R**	**721 NC**	**722 R**	**722 L**	**722 LR**	**722 2R**	**722 NC**
1	0	0	0	0	0	0	0	0	0	0
2										
3										
4										
5	0	0	0	0	0	0	0.5	0	0	0
6	0	0	0	0	0	0	0	0	0	0
7	0	0	0	0	0	0	0	0	0	0
8	0	0	0	0	0	0	0	0	0	0
9										
10										
11	0	0	0	0	0	0	0	0	0	0
12	0	0	0	0	0	0	0	0	0	0
13	0	0	0	0	0.5	0	0	0	0	0
14	0	0	0	1	1	0.5	0	0	0	0
15	0	0	0	1	2	1.5	0	0	0	0
16										
17	0	0	0	1	2	2.5	0	0	0	0
18	0	0	0	0.5	1.5	2.5	0	0	0	0
19	0	0	0	0.5	1	2	0	0	0	0
20	0	0	0	0.5	0.5	2	0	0	0	0
21	0	0	0	0.5	0.5	1.5	0	0	0	0

Figure 3B:

Control										
Day	871 R	871 L	871 LR	871 2R	871 NC	872 R	872 NC	917 R	918 R	919 R
1	2.9	1.4	3.5	2.4	1.4	1.4	-0.5	2.5	1.2	-0.7
2										
3										
4										
5										
6	5.3	11.2	12.9	10.4	3.8	4.1	5.9	6	5.4	6.6
7	5.3	13.1	13.4	11.3	3.8	4.7	6.3	6.4	4.3	9
8	7.7	15	17.3	13.7	5.2	5.4	10.2	7.5	5.4	9.7
9	8.2	14.5	19.8	14.2	5.7	5.4	9.8	7.5	4.7	11.1
10										
11										
12	6.7	4.7	-9.4	17.5	8.1	7.4	-1.5	-11.4	5	11.1
13	5.8	-3.7	-13.9	13.2	5.2	6.8	-11.7	-15.7	1.9	9
14	-2.9	-10.7	-15.3	15.1	8.6	5.4	-16.1	-16.4	-8.1	13.5
15	-10.6	-12.1	-14.9	13.7	9.5	-3.4	-16.1	-14.9	-16.3	13.2
16	-17.3	-14	-16.3	10.4	10.5	-7.4	-15.6	-15.7	-18.6	10.8
17										
18	-17.3	-14	-16.3	-5.7	8.1	-6.8	-18.5	-15.7	-17.4	-8.3
19	-17.3	-14	-16.3	-8	9	-7.4	-21	-15.7	-19.8	-8.3
20	-17.3	-14	-16.3	-7.5	11.4	-5.4	-21	-15.7	-17.1	-8.3
21	-17.3	-14	-16.3	-2.8	2.4	-1.4	-17.1	-15.7	-16.7	-8.3

Control										
Day	490 R	490 NC	491 2R	492 LR	478 L	479 R	479 L	479 LR	479 2R	479 NC
1	0	0.8	0.4	-0.4	1.2	2.5	3.3	-0.6	1.6	3.4
2										
3										
4										
5	2.2	4.2	5.3	0	9	7.1	6.5	7.6	5.8	7.8
6	4	4.6	7.3	0.4	12	8.6	8.7	8.8	7.4	8.9
7	6.2	7.6	7.3	1.6	13.3	13.2	12	11.1	7.9	10.6
8	4.8	5.1	8.2	2.8	13.9	15.2	14.7	11.7	11.6	10.1
9										
10										
11	4	4.6	10.6	1.6	9	10.2	1.1	5.3	10.6	10.1
12	8.8	3.8	6.5	-7.1	-4.2	-4.6	-9.2	-6.4	-3.2	12.8
13	6.6	2.1	-4.1	-15	-9.6	-9.6	-13.6	-5.8	-11.6	7.3
14	7	-0.8	-13.1	-19.4	-7.2	-10.2	-10.9	-4.1	-10.6	-2.2
15	6.2	-6.3	-13.1	-18.6	-7.2	-10.7	-9.8	-4.1	-13.2	-10.1
16										
17										
18	-12.8	-9.7	-16.7	-15	-3	-13.2	-3.3	-11.7	-6.3	-16.2
19	-19.4	-7.6	-14.7	-13.8	-2.4	-10.2	0.5	-12.3	-2.6	-14.5
20	-19.4	-5.9	-12.2	-13.4	0.6	-8.6	2.2	-9.4	-0.5	-14.5
21	-20.7	-4.2	-12.2	-11.5	1.8	-7.6	4.3	-5.8	0.5	-13.4

Control										
Day	725 R	725 L	725 LR	725 2R	725 NC	726 R	726 L	726 LR	726 2R	726 NC
1	0	0	0	0	0	0	0	0	0	0
2										
3										
4										
5	-2.2	1.3	6.3	-2.2	4.8	-0.4	3.1	-2.7	7.3	10.5
6	-0.9	0	7.3	-3.9	1.4	0.4	0.4	-4.6	8.3	7.4
7	-0.4	0	7.8	-3	3.8	-1.3	0	-3.2	8.3	8.3
8	0.4	1.3	10.7	-1.7	2.4	-0.4	4.4	-2.7	10.7	7
9										
10										
11	-3	0	11.7	-4.3	2.9	-5	3.1	-2.3	8.3	3.9
12	-3	2.2	9.7	-1.7	3.3	-4.2	2.7	-0.9	8.7	7.9
13	-5.2	-2.2	-2.9	-3.5	2.4	-5	5.3	-0.5	8.3	7
14	-3.9	-7.6	-5.3	-2.2	6.7	-5	2.7	-0.5	14.1	5.7
15	-0.9	-14.3	-12.1	-4.8	5.3	-1.3	4	3.7	11.7	5.7
16										
17										
18	3.5	-20.2	-8.3	-17.8	5.7	-2.5	3.1	0.5	8.7	5.2
19	5.2	-19.3	-7.3	-17	5.7	-0.8	7.6	0.9	6.8	8.3
20	5.6	-13	-5.3	-14.3	0	-0.8	6.7	0	1	9.6
21	5.6	-13.5	-2.4	-14.8	-5.7	-2.9	4	0.9	-1.9	9.2

GA PLGA-particles; 4 mg/kg; Lot# 1										
Day	869 R	869 L	869 LR	869 2R	869 NC	872 L	917 L	918 L	919 L	919 LR
1	2.7	3.7	1.5	-1.6	3.4	8.4	1.7	1.6	2.6	1.4
2										
3										
4										
5										
6	9.2	11.5	9.7	6.9	5.8	17.7	10	6.2	6.2	2.7
7	11.4	11.5	10.7	8.5	11.2	14.3	9	5.8	7	4.8
8	11.4	12	11.7	10.1	10.7	10.8	10	6.6	7.3	3.4
9	11.4	14.1	9.7	12.8	12.1	11.8	8.7	7.8	7	5.5
10										
11										
12	11.4	5.2	9.7	-11.7	0.5	11.3	9	6.2	4.8	-2.1
13	13.5	7.9	10.2	-14.9	-9.7	-1.5	9.3	5.8	0	-13
14	16.2	9.4	6.1	-14.4	-14.6	-6.4	10.7	3.9	-9.9	-18.2
15	16.8	2.1	-3.1	-14.4	-19.4	-11.3	9.7	-3.9	-15.8	-19.2
16	16.2	-5.2	-10.2	-12.8	-16	-14.8	10.7	-8.2	-18.7	-16.8
17										
18	14.1	-14.7	-14.3	-8	-16	-4.9	12.1	-10.1	-19	-16.4
19	10.3	-15.7	-14.3	-5.9	-14.1	-4.4	11.1	-9.3	-19.4	-16.4
20	6.5	-16.2	-12.2	-5.9	-11.2	-2.5	10	-8.2	-17.9	-17.5
21	2.2	-15.2	-12.2	-3.2	-12.1	-2.5	10.4	-7	-20.9	-14

GA PLGA-particles; 8 mg/kg; Lot# 1										
**Day**	**987 R**	**987 L**	**987 LR**	**987 2R**	**987 NC**	**018 R**	**018 L**	**018 LR**	**018 2R**	**018 NC**
1	0	0	0	0	0	0	0	0	0	0
2	-10.7	-4.4	-1.4	-3.9	-5.5	-2.4	1.4	-10	3.8	-5.1
3	-8.3	-1.9	-1.9	-1.5	-6.8	1.2	2.2	-4	1.5	-3.1
4										
5										
6	-3.9	-1	-0.5	-2	-0.9	2	0.4	-0.7	4.2	-6.8
7	-0.5	0	0.5	-0.5	0.9	2.8	4.3	0.7	6.1	-2.1
8	-0.5	1.5	1.4	-0.5	2.3	5.2	6.5	0.3	3.1	0
9										
10	-1.5	1.9	4.3	2.9	6.8	6.3	4.7	2.3	8.4	1.4
11										
12										
13	-1	3.9	8.7	2.4	1.8	7.1	2.9	5	7.7	2.1
14	-1.5	3.9	9.2	3.9	2.3	13.5	4.3	5.3	9.6	3.4
15	-2	2.4	2.9	3.4	-2.3	8.3	5.8	6.3	8.8	3.4
16	-2.9	1.5	-9.2	2.9	-11.9	5.2	3.2	3	7.7	1.7
17	0	2.9	-12.6	5.4	-11.9	8.7	3.6	4.3	12.3	-0.7
18										
19										
20	0	5.3	-15.9	6.3	-16	9.1	6.1	8.3	11.5	2.7
21	2.4	4.4	-12.6	5.4	-16	6.7	6.1	5	6.1	2.7

GA PLGA-particles; 8 mg/kg; Lot# 2										
**Day**	**719 R**	**719 L**	**719 LR**	**719 2R**	**719 NC**	**720 R**	**720 L**	**720 LR**	**720 2R**	**720 NC**
1	0	0	0	0	0	0	0	0	0	0
2										
3										
4										
5	5.9	-0.5	-5	3.4	8.2	-0.9	2.2	-3.9	5.1	0
6	3.9	0.5	-5.9	2.4	5.6	-0.9	3	-3.4	5.6	1.3
7	6.9	4.1	-5.9	3.4	9.2	0	2.6	-2.1	4.1	3.1
8	4.4	3.6	-3.2	5.3	12.2	1.3	3	0	6.1	3.1
9										
10										
11	4.4	8.6	-1.4	3.4	10.7	1.3	0	0	8.6	3.6
12	5.9	4.5	-1.8	3.9	11.7	1.7	-4.3	1.7	5.1	3.6
13	4.9	-8.1	-1.4	3.4	13.3	-0.9	-11.7	-5.2	5.1	3.1
14	6.9	-16.7	-11.8	4.4	14.3	1.3	-11.7	-14.6	5.1	4.9
15	8.3	-20.8	-18.1	-5.8	14.3	1.3	-9.5	-19.7	-1.5	0
16										
17	14.7	-17.2	-18.6	-12.1	12.2	2.6	-6.1	-21	-5.6	-13.4
18	12.7	-14	-17.2	-12.1	12.2	3.9	-6.1	-18.5	-10.7	-17.9
19	12.3	-11.3	-14.5	-6.8	18.4	4.8	-6.5	-20.2	-12.2	-15.2
20	5.4	-10.4	-14	-5.8	16.8	4.8	-9.5	-20.2	-14.2	-15.2
21	-1	-10.4	-13.1	-3.9	18.4	4.3	-8.2	-20.2	-15.2	-15.2

KGYY$_6$PLGA particles; 8 mg/kg; Lot# 1										
**Day**	**807 R**	**807 L**	**807 LR**	**807 2R**	**807 NC**	**810 L**	**810 LR**	**831 NC**	**832 R**	**832 L**
1	0	0	0	0	0	0	0	0	0	0
2	0	0	0	0	0	0	0	0	0	0
3	0	0	0	0	0	0	0	0	0	0
4	0	0	0	0	0	0	0	0	0	0
5	4.5	1.5	5.6	6	6.1	-2.9	5.4	1.6	1.8	1.1
6	6.4	5.3	8.7	5.5	7.1	-3.9	5.4	1.2	1.4	1.4
7	6.8	6.3	8.7	7.1	7.6	-1.5	6.3	5	-0.4	2.1
8	6.8	6.8	7.2	8.8	8.6	-2	7.3	3.5	0	0.7
9										
10										
11	9.1	3.9	7.7	6	7.1	0.5	6.3	4.7	2.1	3.9
12	6.4	3.4	7.2	4.9	5.6	-2.9	6.8	5.4	0.7	1.8
13	7.3	1.5	9.2	6	6.1	-4.4	7.8	5.4	1.8	0.4
14	8.2	4.4	11.3	4.9	6.6	-3.4	-1	0.4	1.4	0
15	9.1	6.8	11.8	6	9.1	2.4	-7.3	-6.2	1.1	1.4
16	10	9.2	9.7	7.1	9.6	3.9	-10.7	-15.5	-6.7	1.8
17	7.3	7.3	8.7	2.7	6.1	3.9	-10.2	-14.3	-15.1	0.7
18	8.2	8.7	13.3	7.1	6.6	2	-6.8	-12.4	-12	3.6
19	8.2	6.8	14.4	4.9	7.6	1.5	-5.9	-14.7	-10.6	2.8
20	10.5	7.8	16.4	6	9.6	6.3	-2.9	-13.2	-7.4	1.4
21	11.4	6.8	15.4	6.6	9.6	6.8	-2	-9.3	-4.9	4.6

KGYY$_6$PLGA particles; 8 mg/kg; Lot# 2										
**Day**	**721 R**	**721 L**	**721 LR**	**721 2R**	**721 NC**	**722 R**	**722 L**	**722 LR**	**722 2R**	**722 NC**
1	0	0	0	0	0	0	0	0	0	0
2										
3										
4										
5	2.9	5.4	7.8	-0.5	4.3	7.3	8.7	1.4	-2.3	0.4
6	0	5.9	5.2	-1	4.3	8.8	7.7	0.9	-0.5	0.9
7	1.9	5	6.9	-2.4	6	8.3	7.2	0.5	1.4	2.2
8	4.8	8.4	7.3	-1	4.7	11.7	7.7	0	4.5	3
9										
10										
11	2.9	4.5	5.2	-1	0	10.7	4.8	-2.8	1.4	0.4
12	5.7	9.9	6.9	0	-3.9	12.7	6.8	0	2.3	3
13	4.3	9.4	5.2	-2.9	-13.3	10.7	6.3	-0.9	0.9	1.7
14	6.7	8.9	8.6	-2.4	-20.2	6.8	7.7	-1.4	4.1	6.1
15	7.1	6.9	13.8	-2.9	-15.9	-5.4	8.2	-0.9	5.5	9.5
16										
17	9	8.4	15.9	-3.3	-15	-10.2	10.6	1.9	6.8	10.4
18	7.1	7.9	17.2	-2.9	-10.3	-6.3	8.2	1.9	6.4	12.6
19	10	9.4	18.5	-0.5	-8.6	-2.4	10.1	2.8	7.7	12.1
20	8.1	7.4	19.4	-1.4	-7.3	-2	10.1	0.9	4.5	14.7
21	8.1	6.9	17.7	-1	-9.4	-2	10.6	0.5	6.4	13
